# Hematogenous vertebral osteomyelitis caused by vaginal microbiota: metagenomic resolution of a polymicrobial anaerobic case

**DOI:** 10.1007/s15010-026-02926-4

**Published:** 2026-08-18

**Authors:** Isabelle Vock, Annika Bargetzi, Maja Weisser, Olivia Katharina Mueller, Milena Junker, Arne Mehrkens, Claudio Neidhoefer, Baptiste Hamelin, Salome Hosch, Kirsten D. Mertz, Peter M. Keller, Richard Kuehl

**Affiliations:** 1 Division of Infectious Diseases, University Hospital Basel, Basel, Switzerland; 2 Department of Internal Medicine, University Hospital Basel, Basel, Switzerland; 3 Division of Spine Surgery, University Hospital Basel, Basel, Switzerland; 4 Clinical Bacteriology and Mycology, University Hospital Basel, Basel, Switzerland; 5 Institute of Medical Genetics and Pathology, University Hospital Basel, Basel, Switzerland; 6 Center for Infection Diagnostics, University Hospital Basel, Basel, Switzerland; 7 Center for Musculoskeletal Infections, University Hospital Basel, Basel, Switzerland

**Keywords:** Vertebral osteomyelitis, Spondylodiscitis, Vaginal microbiota, Anaerobic bacteria, Metagenomic next-generation sequencing, 16S rRNA sequencing, *Fannyhessea vaginae*, *Gardnerella vaginalis*

## Abstract

We describe a rare case of a 35-year-old female patient suffering from polymicrobial hematogenous vertebral osteomyelitis caused by vaginal microbiota following sexual intercourse. Anaerobic blood cultures yielded *Fannyhessea vaginae* and *Gemelliphila asaccharolytica*, and intraoperative tissue cultures from decompression surgery identified *Gardnerella vaginalis*. Beyond *Fannyhessea vaginae* and *Gemelliphila asaccharolytica*, 16S rRNA gene Nanopore sequencing of surgical tissue also detected high amounts of *Parvimonas parva*,* Peptostreptococcus anaerobius*, *Marseillibacter massiliensis*,* and Gemelliphila palaticanis*. Antibiotic treatment with broad anaerobic coverage resulted in complete clinical resolution. Retrospective metagenomic analysis of a cervical swab obtained 9 months earlier revealed *Fannyhessea vaginae* and *G. vaginalis* to be already present in the vaginal microbiota. This case highlights the potential for hematogenous dissemination of vaginal anaerobes after sexual intercourse and underscores the diagnostic challenges posed by fastidious anaerobic bacteria. Molecular techniques are helpful tools in uncovering pathogens that may escape conventional culture methods.

## Background

Vertebral osteomyelitis (VO) is a common infection of the spine with increasing incidence globally within the past decades [1]. *Staphylococcus aureus* still accounts for a majority of bacterial VO [2]. Anaerobic bacteria have been described in less than 2% of VO, with a predominance in female patients, postulated to be associated with the high proportion of anaerobic bacteria in the vaginal flora [3]. VO due to vaginal bacteria is a rarity and has been described in only few case reports [4]. Genitourinary procedures, inlaying genitourinary foreign materials and mucosal injuries have been associated with extra genitourinary infections caused by vaginal bacteria, leading to hematogenous bacterial dissemination [4, 5]. Only limited data on risk factors and optimal management are available.

The aim of this report is to describe a case of polymicrobial hematogenous VO caused by vaginal microbiota in a sexually active female person without prior gynecological intervention and furthermore to discuss the added diagnostic value of molecular techniques in detecting fastidious pathogens [6].

## Case description

A 35-year-old female presented to the emergency department with a two-day history of progressively worsening, immobilizing lumbar pain radiating to both lower extremities. The patient reported sexual intercourse two days before presentation and noted increased foul-smelling vaginal discharge thereafter. Her medical history was notable for intravenous drug use, VO caused by *Pseudomonas aeruginosa* nine years earlier, and bacterial vaginosis with detection of *Gardnerella vaginalis* from a cervical swab nine months earlier.

**Fig. 1:**
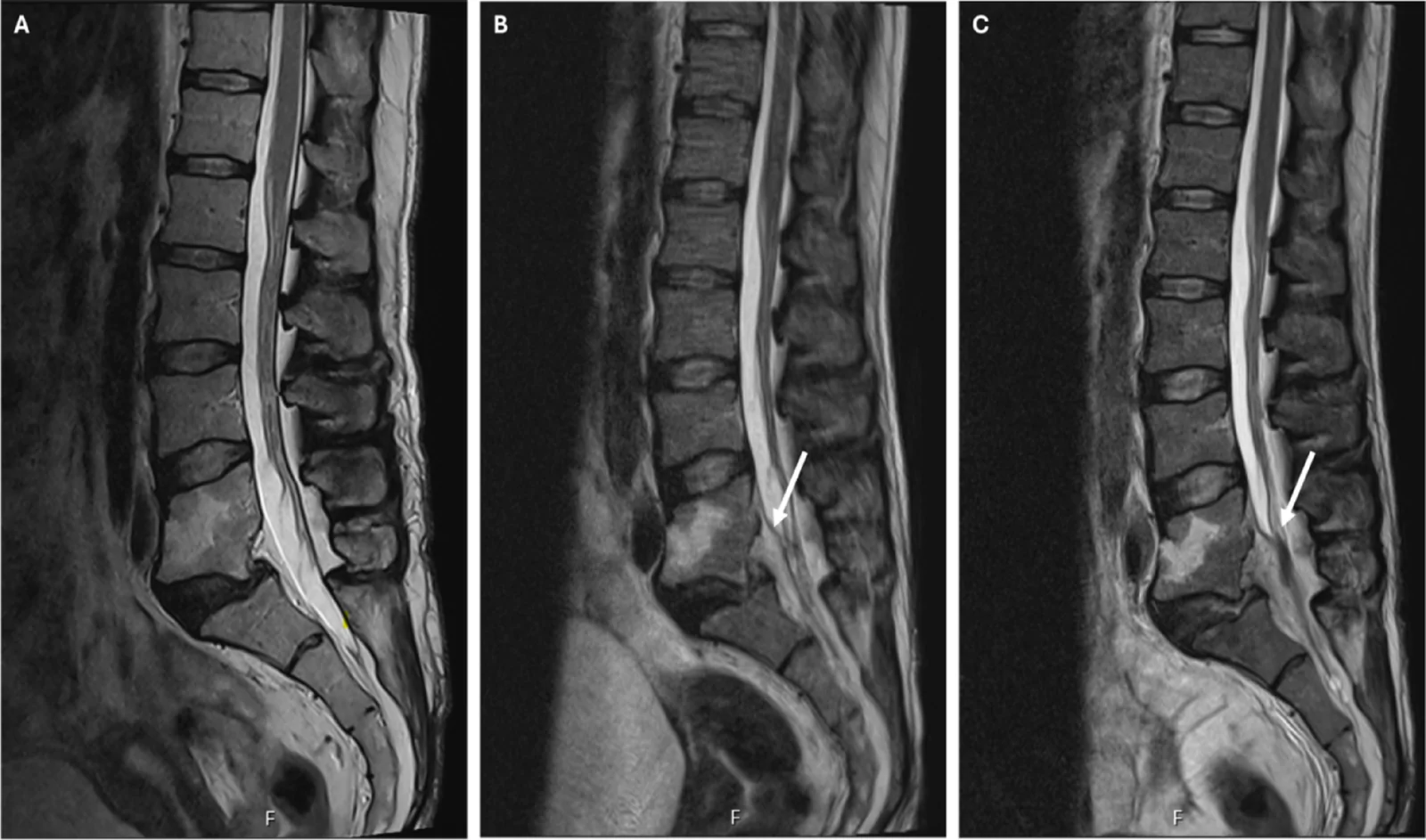
MRI scans of the spine initially showed no signs of vertebral osteomyelitis (**A**) while follow-up imaging revealed spondylodiscitis at L5/S1 (**B**) with an increasing epidural abscess (**C**)

On admission, she was afebrile (37.0 °C), normotensive (122/84 mmHg) and had a normal heart rate (77 bpm). The lumbar spine was tender to palpation. Neurologic examination was unremarkable, and no cutaneous stigmata of endocarditis were observed. Gynecological pelvic examination revealed no pathological findings, in particular no clinical signs of pelvic inflammatory disease (PID). Initial laboratory workup demonstrated elevated C-reactive protein (CRP) levels (51 mg/L). No blood cultures were obtained at admission. MRI of the lumbar spine confirmed previously detected high-grade foraminal stenosis but showed no signs of vertebral osteomyelitis (Fig. 1 A). Three days after admission, the patient’s condition deteriorated, with progressive lumbar pain, new-onset fever, chills, and rising CRP levels (298 mg/L). Blood cultures obtained before initiating empiric intravenous amoxicillin–clavulanate yielded *Fannyhessea vaginae* in both anaerobic bottles within 44 h. Additionally, vaginal swab cultures grew *Gardnerella vaginalis*. Polymerase chain reaction (PCR) for *Chlamydia trachomatis* and *Neisseria gonorrhoae* of the vaginal swab was negative, as were serologic tests for HIV and syphilis.

**Fig. 2:**
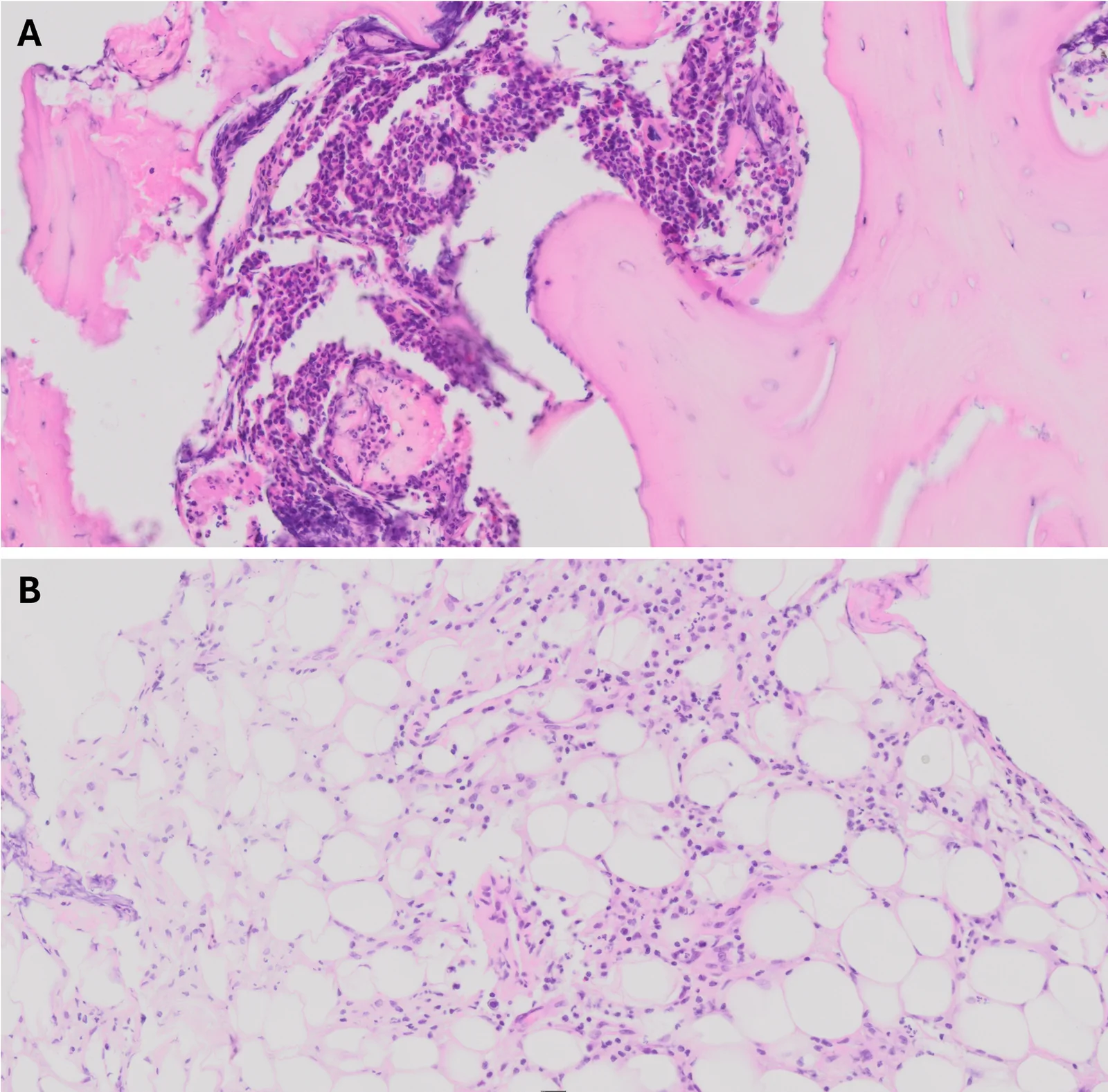
Histological images exhibiting florid inflammation in vital corticospongious bone tissue (**A**) along with fatty and connective tissue (**B**)

Given ongoing clinical suspicion of VO, repeat lumbar spine MRI revealed spondylodiscitis at L5/S1 with an epidural abscess causing severe spinal stenosis (Fig. 1B). Transthoracic and transesophageal echocardiography was performed to rule out infective endocarditis. The patient underwent microsurgical decompression and drainage. Intraoperative tissue cultures yielded *Gardnerella vaginalis*. Histopathology showed florid inflammation in viable corticospongious bone tissue (Fig. 2 A), and in fatty and connective tissue (Fig.2B). Subsequently, the initially drawn blood cultures showed slowly growing Gram-negative anaerobic cocci. Species identification by MALDI-TOF mass spectrometry was inconclusive, whereas 16S rRNA gene sequencing identified *Gemelliphila asaccharolytica*. Results of antimicrobial susceptibility testing are shown in Table 1. Given the diagnosis of VO caused by vaginal microbiota distinct from the bacteria isolated from blood cultures, 16S rRNA gene (nanopore) sequencing was also applied to unfixed lumbar tissue samples for broad bacterial species identification and taxonomic classification, revealing a polymicrobial vaginal bacterial flora as shown in Table 1. Further, metagenomic next-generation sequencing (mNGS) was performed on formalin-fixed paraffin-embedded (FFPE) lumbar tissue samples and on the vaginal swab obtained one year before, detecting high read counts of *Fanyhessea vaginae* and *Gardnerella vaginalis* at both sites. Due to persistent fever following surgical revision, metronidazole (500 mg every 8 h) was added to broaden antibiotic coverage of anaerobic bacteria as we hypothesized potential susceptibility of culture-negative anaerobes, leading to rapid clinical improvement. The patient was discharged after 19 days and completed a six-week course of oral amoxicillin-clavulanate and metronidazole, resulting in full clinical recovery.

## Discussion

Our case illustrates the potential for hematogenous dissemination of vaginal anaerobes following sexual intercourse and underscores the diagnostic challenges posed by fastidious anaerobic bacteria. The clinical presentation of our patient is typical for VO, characterized by acute lumbar pain and elevated inflammatory markers. Intravenous drug use is a known risk factor for VO [7] and is usually caused by bacteria of the skin flora, which initially guided the differential diagnosis. However, our case raises awareness to consider atypical pathogens in sexually active women, especially in the presence of concurrent or preceding vaginal symptoms.

The anaerobic bacteria *G. vaginalis* and *F. vaginae* can asymptomatically colonize the human genitourinary tract [8] and have been associated with bacterial vaginosis (BV) [9]. Our patient had a history of prior BV with *G. vaginalis* detected in culture and *F. vaginae* identified retrospectively by mNGS of the corresponding specimen, suggesting chronic genitourinary colonization. Since the patient did not report any genitourinary interventions, we hypothesize that sexual intercourse might have induced microtrauma of the vaginal mucosa, which combined with bacterial vaginosis facilitated hematogenous dissemination of the vaginal microbiota. A similar case of a spondylodiscitis caused by a polymicrobial vaginal flora in an immunocompetent female patient was reported by Kolakowska et al. [10], where sexual intercourse was likewise suspected as potential source.

While molecular techniques greatly enhanced pathogen detection in this case, several limitations must be acknowledged. Differentiating true pathogens from commensals or contaminants remains challenging, particularly when organisms are detected solely by sequencing and were not recovered in culture. In our case, several anaerobes (e.g., *Gemelliphila palaticanis*, *Peptostreptococcus anaerobius*) have not been previously reported as human pathogens, and their clinical relevance is therefore uncertain. Low-biomass samples such as vertebral tissue are prone to contamination; we therefore included negative controls (extraction blanks and no-template controls) in both the Nanopore 16S rRNA sequencing and mNGS workflows, with no relevant reads mapping to the organisms of interest.

Retrospective metagenomic analysis of the vaginal swab obtained 9 months earlier demonstrated the presence of *Fannyhessea vaginae* and *Gardnerella vaginalis*, supporting chronic colonization. However, the vaginal microbiota is highly dynamic, so this does not prove identical composition at the time of bacteremia. Quantitative metrics (high read counts and relative abundance of key organisms in both tissue and vaginal samples) further support their relevance. Nevertheless, causality ultimately relies on integration with clinical, microbiological, and histopathological findings rather than sequencing data alone.

In our case, unbiased metagenomic analysis of spinal tissue revealed several additional anaerobic bacteria, likely also originating from the vaginal microbiota, consistent with the clinical scenario and known reservoirs of these bacteria. *Gemelliphila asaccharolytica* are Gram-variable, facultatively anaerobic cocci which have been described as part of the vaginal microbiome [11]. *Marseillibacter massiliensis* and *Parvimonas parva* are Gram-positive anaerobes, that have been isolated in human stool and urinary samples, respectively [12, 13]. To date, *Gemelliphila palaticanis* and *Peptostreptococcus* anaerobius have not been reported in human clinical samples but have been identified in zoonotic reservoirs [14, 15]. The simultaneous hematogenous translocation of multiple anaerobic bacteria to a distant focus suggests transmission of multispecies biofilm aggregates, as known to occur in vaginal infections [16], potentially protecting bacteria from immune-mediated clearance. However, the clinical relevance of these anaerobes detected solely by sequencing methods in the vertebral tissue samples remains uncertain. Nevertheless, the patient clinically improved following the addition of metronidazole, despite reported metronidazole resistance of *G. vaginalis* and *G. asaccharolytica*, suggesting an additive therapeutic effect targeting strictly anaerobic bacteria.

Diagnosing infections with difficult-to-culture pathogens such as anaerobes or antibiotic pre-treated bacteria remains challenging [17]. Novel diagnostic approaches such as metagenomic sequencing can provide additional diagnostic yield in uncovering pathogens that may escape conventional culture methods and are less dependent on prior antibiotic therapy [18]. In our case, metagenomics complemented standard diagnostics and resolved discrepant culture results of blood and spinal tissue. The detection of *G. vaginalis* in culture but its near-absence via 16S rRNA sequencing (4 reads) in the vertebral biopsies can be explained by an exceptionally low initial bacterial load, yielding only 2 colony-forming units. A recent retrospective study reported a sensitivity of 70.3% and a specificity of 75% for metagenomic Next Generation Sequencing in spinal infections, exceeding tissue cultural sensitivity by 14.8% [19]. The Infectious Diseases Society of America recommends molecular diagnostics in VO as part of an extended diagnostic workup, particularly in culture-negative patients [20]. Here, we performed metagenomic analysis on both fresh and FFPE samples according to previously published protocols and sequencing platforms [21]. Negative controls were always included and ruled out vaginal contaminants. Analysis of FFPE material enables retrospective testing of conserved histology specimens [21] and, in this case, supported causality by detecting the same anaerobes in one-year old fixed vaginal samples and in the VO.

However, the application of advanced molecular diagnostic methods requires careful interpretation. Differentiating true pathogens from commensals or contaminants remains challenging and may lead to overtreatment and unnecessary healthcare costs. Conversely, accurate identification of causative organisms facilitates targeted antimicrobial therapy, promoting antimicrobial stewardship efforts, and may potentially reduce the need for extensive diagnostic procedures. Interdisciplinary case discussions including infectious diseases specialists, microbiologists and pathologists are therefore crucial for optimal diagnostic and therapeutic decision-making.

In conclusion, this case highlights the potential of vaginal microbiota to cause hematogenous vertebral osteomyelitis and emphasizes the value of adding molecular diagnostic approaches to improve diagnostic accuracy.

**Table 1:** bacterial species identified by different diagnostic methods and minimal inhibitory concentrations (MICs) to listed substances of culture-positive isolates (mg/L); number in parentheses next to antimicrobial agents indicate clinical susceptibility breakpoints for susceptible (S) and resistant (R) isolates (mg/L), according to CLSI criteria; no established breakpoints were available for Vancomycin; blank MIC fields signify “not tested”; mNGS = metagenomic Next-Generation Sequencing

Material	Method	Pathogens			
		*Gardnerella vaginalis*	*Fannyhessea vaginae*	*Gemelliphila asaccharolytica*	*Gemelliphila palaticanis Marseillibacter massiliensis Parvimonas parva* *Peptostreptococcus anaerobius*
Blood	Culture		X	X	
Vaginal swab	Culture	X			
	mNGS	X	X		
Vertebral biopsies	Culture	X			
	Nanopore 16S rRNA sequencing	X (4 reads)	X (7497 reads)	X (9018 reads)	X (1246; 1135; 8460; 1458 reads)
	mNGS	X	X		
*Minimal inhibitory concentrations*					
Penicillin (< 0.5 S; ≥2R)		0.125 mg/L	0.094 mg/L	0.004 mg/L	-
Clindamycin (< 2 S; ≥8R)		0.047 mg/L	< 0.016 mg/L	0.094 mg/L	-
Vancomycin		0.25 mg/L	0.5 mg/L	0.125 mg/L	-
Metronidazol (≤ 8 S; ≥32R)		32 mg/L	-	> 256 mg/L	-

## Data Availability

No datasets were generated or analysed during the current study.
